# Reactions of polyphenols in pomegranate peel with nitrite under simulated stomach conditions

**DOI:** 10.1002/fsn3.1173

**Published:** 2019-08-21

**Authors:** Zhenjian Xie, Xiaohong Li, Renyong Tang, Guoze Wang, Yurong Lu, Xuemei Li, Kun Cheng, Linzhi Li, Qiang He

**Affiliations:** ^1^ College of Pharmacy and Biological Engineering Chengdu University Chengdu China; ^2^ College of Light Industry and Food Engineering Sichuan University Chengdu China

**Keywords:** ellagic acid, nitrite, nitrosation, polyphenols, pomegranate peel, punicalagin

## Abstract

Punicalagin and ellagic acid are the major polyphenols present in pomegranate peels. The contents of α‐punicalagin, β‐punicalagin, and ellagic acid in the pomegranate peels were approximately 75, 72, and 20 µM, respectively. The reactions of polyphenols in pomegranate peels with sodium nitrite under simulated stomach conditions were studied. The reactions decreased the polyphenolic contents of the pomegranate peels and accompanied the formation of nitroso compounds. The oxidation rates followed the order ellagic acid ＜α‐punicalagin ≈ β‐punicalagin. The results suggested that the reactions can occur in the stomach after a meal, while the pH changes from 2 to 4.5.

## 1 INTRODUCTION

**Figure 1:**
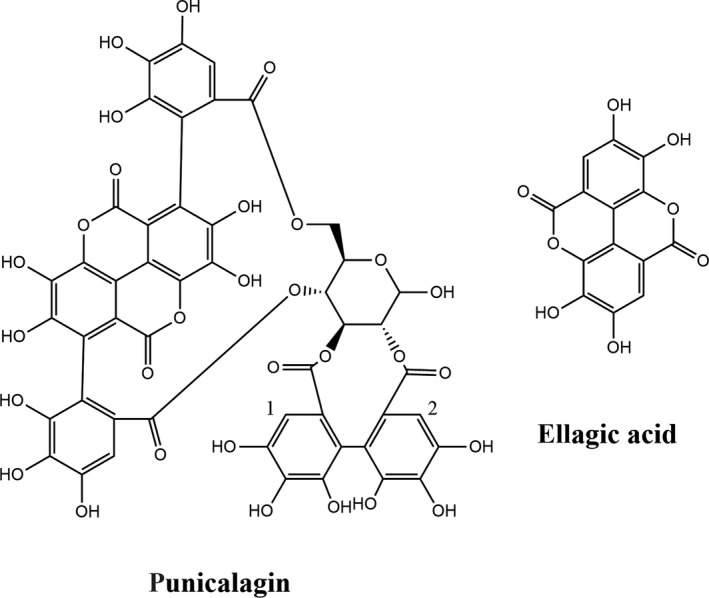
The structures of punicalagin and ellagic acid

Pomegranate (Punica granatum L.) is deciduous shrubs or dungarunga, which is located in tropical and subtropical regions (Du, Li, Zhang, Wang, & Zhang, 2018). The pomegranate peel is a by‐product that composes approximately half of the whole fruit (Al‐Said, Opara, & Al‐Yahyai, 2009). It is a traditional ingredient in Asian cuisine. Pomegranate peel extracts have a potential to be used as food additives (Akhtar, Ismail, Fraternale, & Sestili, 2015). It is well known that the pomegranate peel contains more abundant polyphenols than the juice, seed, and leaves. Punicalagin and ellagic acid (Figure 1) are the major polyphenols in pomegranate peel polyphenols (PPPs), and the content of punicalagin may reach 10.50 to 98.00 mg/g of the total polyphenols in the pomegranate peels (Carbone, Garrigós, & Jiménez, 2016). Pomegranate peel extracts have been proven to have significant antioxidant capacities in many studies. (Derakhshan et al., 2018; Šavikin et al., 2018). In addition, it has many biological activities such as antibacterial, tyrosinase‐inhibition (Fawole, Makunga, & Opara, 2012), anti‐inflammation (Du et al., 2018; Verotta et al., 2018), antiproliferative activity (Masci et al., 2016), anti‐diabetic (Šavikin et al., 2018; Stojanović et al., 2017), and anti‐cancer activity (Dikmen, Ozturk, & Ozturk, 2011). Moreover, pomegranate polyphenol dietary supplements have also been confirmed by in vitro and in vivo studies as highly safe (Heber et al., 2007; Vidal et al., 2003).

It is well known that dimethylnitrosamine (NDMA) and diethylnitrosamine (NDEA) are carcinogenic, mutagenic, and teratogenic (Choi & Valentine, 2002). They have been commonly detected in cured meat, cured fish, and pickled vegetables and can be synthesized in vivo. Nitrite, as the immediate precursor may react with secondary amines under the acidic conditions of the stomach, potentially leading to digestive cancer (Choi, Chung, Lee, Shin, & Sung, 2007). At acidic pH, nitrite may be present as nitric oxide (•NO) and other nitrogen oxides with nitr(os)ating activity (Ferreira et al., 2011). The nitrosation of some phenolic compounds such as catechin, rutin, quercetin, caffeic acid, and chlorogenic acid (Hirota & Takahama, 2015; Lee et al., 2006; Morina, Takahama, Mojovic, Popovic‐Bijelic, & Veljovic‐Jovanovic, 2016; Peri et al., 2005; Takahama, Yamauchi, & Hirota, 2013; Takahama, Yamauchi, & Hirota, 2016) has been reported under the conditions simulating the stomach. The reduction of nitrous acid to NO by the above components has been investigated in acidic buffer solutions and acidified saliva.

This report explores and analyzes the reactions of polyphenols in pomegranate peel with nitrite under simulated stomach conditions to discuss the functions of pomegranate peels.

## 2 MATERIALS AND METHODS

### 2.1 Reagents and material

Pomegranate fruits were purchased from the local market of Chengdu City, Sichuan Province, China. The peels were manually separated by hand from the seeds, and then rinsed with distilled water. After being dried in an oven with air circulation at 40°C, the peels were grounded in a laboratory grinder. Particles with a particle size of 0.42 mm were screened according to China Pharmacopoeia.

Ellagic acid and punicalagins were purchased from Sigma‐Aldrich. The other chemicals were purchased from Kelong Chemical Co, Ltd. All the chemical reagents were analytical grade except for the acetonitrile, which was chromatographic grade. All solutions were prepared in deionized water.

### 2.2 Nitrite‐induced oxidation of polyphenols

Pomegranate peel powder (1.0 g) was sonicated in distilled water (250 ml) for 30 min at 40°C. Then, the pomegranate peel extract was centrifuged at 7,000 *g* for 10 min, and the pH was adjusted to 2.0 by adding 2 M HCl. Then, the solution was divided into 1 ml test tubes, different concentrations of sodium nitrite were added to the solutions, and the solutions were placed into a thermostat water bath for incubation. After the incubation, 5 ml of ethyl acetate was added to each incubated sample. The ethyl acetate extract was evaporated under nitrogen in a water bath, and the residue was dissolved in 1 ml mobiles phases. The solutions were centrifuged at 10,000 *g* for 2 min and then used for HPLC analysis.

### 2.3 HPLC analysis

A reverse‐phase HPLC system (Agilent 1260; Agilent Technologies Inc), which used a Diamonsil C18 column (25 cm × 4.6 mm i.d. 5 µm) (Beijing Dikma Co) and a spectrophotometric detector with a photodiode array (G1316A), was employed. The mixtures of 0.2% (v/v) acetic acid (A) and acetonitrile (B) were used as mobile phases. Gradient elution was performed according to the following scheme: 0–5 min, 95% A; 5–15 min, 95%–75% A; 15–25 min, 75%–10% A; 25–30 min, 10%–95% A, with a posttime of 3 min, and the flow rate was 1 ml/min. Polyphenols of pomegranate peel and the reaction products were detected at 378 nm.

### 2.4 LC/MS

Electrospray ionization mass spectra (ESI‐MS) were obtained on an Agilent 6410 Triple Quad LCMS instrument (Agilent Technologies Inc). Samples were separated by a Poroshell 120 EC‐C18 column (100 × 2.1 mm i.d. 2.7 µm) (Agilent Technologies Inc) and then delivered into the ion source. The composition of the mobile phase was consistent with that analyzed by HPLC. The flow rate was 0.3 ml/min.

### 2.5 Statistical analysis

All experiments were repeated in triplicate. The data are presented as the mean ± *SD*.

## 3 RESULTS

### 3.1 Concentration of Polyphenols in pomegranate peels

**Figure 2:**
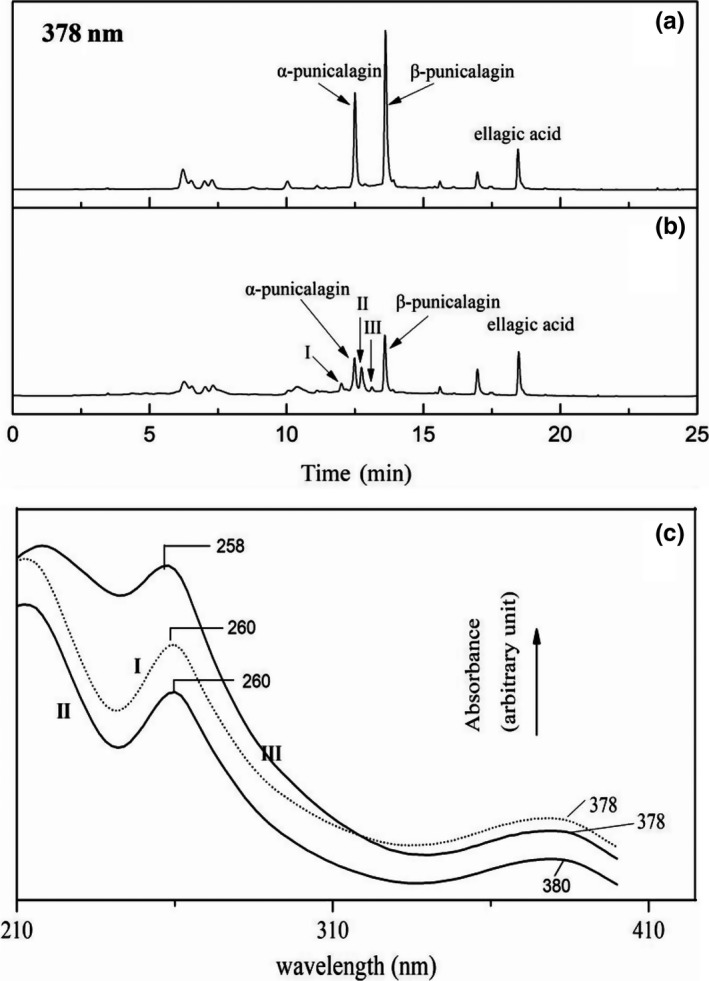
HPLC chromatogram and UV‐visible absorption spectrogram of polyphenols in pomegranate peel. Pomegranate peel extracts were incubated at 37°C for 30 min (a) without sodium nitrite; (b) with 0.5 mM sodium nitrite. (c) UV‐visible absorption spectrogram of the production Ⅰ, Ⅱ, and Ⅲ

Figure 2a (378 nm) shows the typical HPLC chromatogram of a pomegranate peel aqueous extract. The components eluted at approximately 12.5, 13.7, and 18.6 min were identified to be α‐punicalagin, β‐ punicalagin, and ellagic acid by comparing their absorption spectra and retention times with standard compounds. The recognition result was verified by ESI‐MS in negative mode: α‐punicalagin and β‐ punicalagin, *m/z* at 1,083([M‐H]^−^), and ellagic acid *m/z* at 301([M‐H]^−^).These components were reported previously (Çam & Hışıl, 2010; Živković, Šavikin, Janković, Ćujić, & Menković, 2018). Under the HPLC conditions, α‐punicalagin, β‐ punicalagin, and ellagic acid were separated as a major component. The concentrations of the above polyphenols were calculated using calibration curves. The results are presented as micromoles per gram of dry weight (µM/g dw) (Table 1). The mean concentrations of ellagic acid were much lower than those of α‐punicalagin and β‐punicalagin. The presence of these polyphenols in pomegranate peels has been reported in many previous studies (Akhtar et al., 2015; Mushtaq, Sultana, Anwar, Adnan, & Rizvi, 2015).

**Table 1:** Polyphenols in pomegranate peel and their reactions with nitrite

Compounds	Concentration (µM/g)^b^	Rate^a^ (µM g^−1^ hr^−1^)^b^
α‐punicalagin	75.41 ± 4.76	93.77 ± 10.87
β‐punicalagin	72.41 ± 4.96	89.77 ± 10.15
ellagic acid	20.66 ± 1.54	5.69 ± 3.05

a Calculated from the data after 30 min incubation in the presence of 0.5 mM nitrite.

b Dry weight basis.

### 3.2 Reactions of pomegranate peel polyphenols with nitrite

It has been reported that the concentration of salivary nitrite is approximately 0.05 to 1.0 mM during the stay in the stomach (Pannala et al., 2003). The pomegranate peel extract was incubated with sodium nitrite (0.1–1.0 mM) for 30 min. The color turned yellow, and as the concentration of sodium nitrite increased, the color gradually deepened to brown, which was the same as the reactions of polyphenols in masticated apple fruit with nitrite (Hirota & Takahama, 2015).

Pomegranate peel extracts were incubated with 0.5 mM sodium nitrite for 30 min. The typical HPLC chromatogram of the reaction products is shown in Figure 2b (378 nm). The concentrations of α‐punicalagin and β‐punicalagin decreased remarkably, but the decrease in the concentration of ellagic acid was slight. The decreases in polyphenol concentrations were accompanied by the production of I, II, and III, whose retention times were 12.1 min, 12.7 min, and 13.1 min, respectively. The data indicate that product II is the main product. Figure 2c shows the UV/visible absorption spectra of the three productions. The spectrum of product Ⅰ was essentially the same as that of products Ⅱ and Ⅲ, which had an absorption peak at approximately 260 nm and a shoulder at approximately 378nm. This indicates that the three components were supposed to be isomers. The ESI‐MS data displayed the molecular ion at *m/z* 1,117 and the fragment ions at *m/z* 1,055, *m/z* 781 (punicalin moiety) and *m/z* 601 (gallagic acid moiety).

### 3.3 Effect of nitrite concentration and reaction time on the consumption of punicalagin and ellagic acid

**Figure 3:**
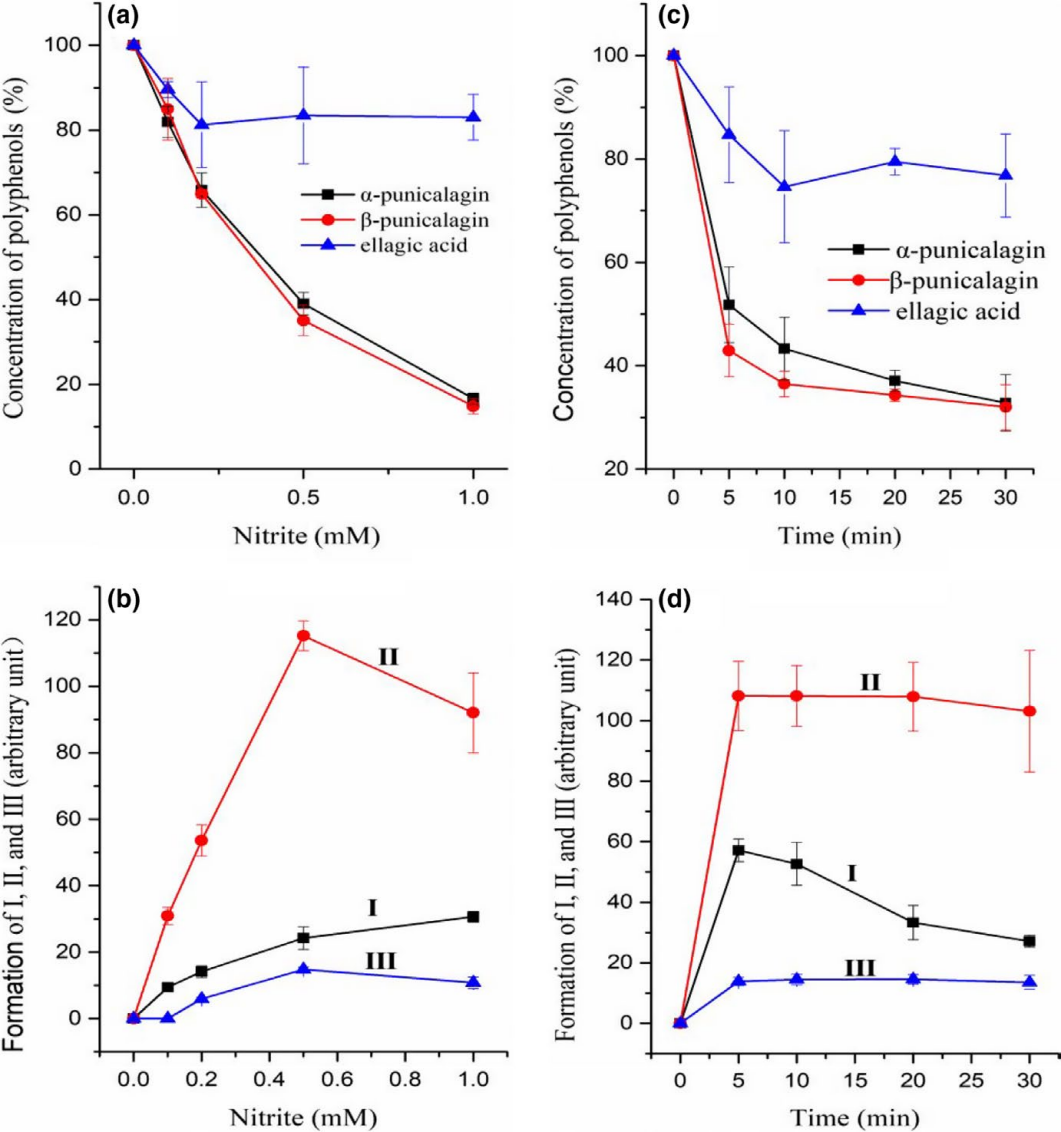
Effects of nitrite concentrations and time on the reactions of pomegranate peel polyphenols with nitrite. (a) and (b) Consumption of pomegranate peel polyphenols and formation of production Ⅰ, Ⅱ, and Ⅲ. The pomegranate peel extracts were incubated at 37°C and pH 2.0 for 30 min with 0–1.0 mmol NaNO_2_; (c) and (d) Time courses of the polyphenols decreasing and the productions forming. The pomegranate peel extracts were incubated with 0.5 mmol NaNO_2_ at 37°C and pH 2.0

Figure 3a shows the changes in α‐punicalagin, β‐ punicalagin, and ellagic acid with increasing nitrite concentrations. The results indicated that with increasing nitrite concentration, the concentrations of α‐punicalagin, β‐ punicalagin, and ellagic acid decreased gradually. In addition, α‐punicalagin and β‐ punicalagin reacted more rapidly and easily with nitrite than ellagic acid. The data in Table 1 also confirmed this result.

Figure 3b shows the formation of I, II and III as the nitrite concentration changed. The formation of I and III increased with increasing nitrite concentrations. When the concentration of the nitrites was lower than 0.5 mM, product II increased rapidly, and the formation of II was nearly linear with the increase of the concentration of nitrites. Then, the nitrite concentration decreased from 0.5 to 1.0 mM.

Figure 3c shows time courses of consumption of α‐punicalagin, β‐ punicalagin, and ellagic acid. These results also indicated that α‐punicalagin and β‐punicalagin reacted with nitrite much more rapidly than ellagic acid. The time course of the decrease in the concentration of α‐punicalagin bore great similarity to that of the decrease in the concentrations of β‐punicalagin. The reactions of α‐punicalagin and β‐punicalagin with nitrite were rapid, while almost half the reactions occurred within 5 min. Time courses of their formation also showed that the reactions were rapid (Figure 3d). The concentration of the products improved in a short time frame and then maintained a steady state.

### 3.4 Effect of pH on the reactions of pomegranate peel polyphenols with nitrite

**Figure 4:**
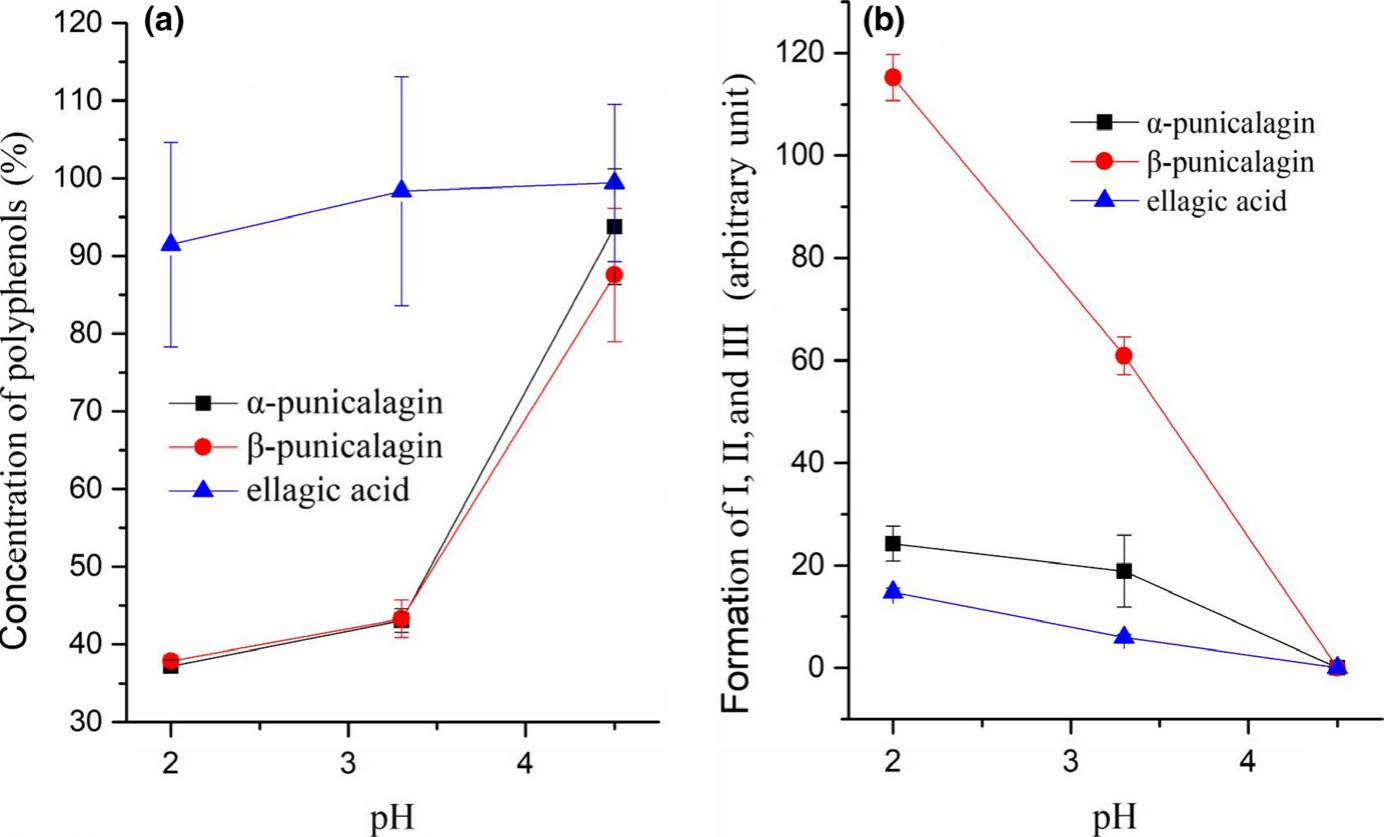
Effects of pH on the reactions of pomegranate peel polyphenols with nitrite

Gastric pH increases from approximately 2～4.5 while eating a meal. Approximately 0.5～1 hr is needed to decrease the pH to below 4, and 1～1.5 hr to approximately 2 after eating (Robinson, 2002). As shown in Figure 4, pomegranate peel polyphenols reacted easily with the nitrites at pH 2. As the pH value increased, the reaction rate decreased, and almost no reactions were observed at pH 4.5. Nitrites decreased the concentrations of α‐punicalagin and β‐punicalagin remarkably compared to ellagic acid. The results suggest that the reactions can occur in the stomach after a meal, while the pH changed from 2 to 4.5. Nitrous acid (pK_a_ = 3.3) may be one of the reasons for the reactions between nitrite and pomegranate peel polyphenols.

## 4 DISCUSSION

Punicalagin, the main ingredient of polyphenol from pomegranate peels. The content of punicalagin may reach 88.70 to 118.60 mg/g dry weight extract from different regions (Khalil, Khan, Shabbir, & Rahman, 2017), while Carbone (Carbone et al., 2016) reported the content of punicalagin in the pomegranate peels ranged from 10.50 to 98.00 mg/g of the total polyphenols.

Nitrates in the body come mainly from green leafy vegetables in the diet and their own synthesis (Hsu, Arcot, & Alice Lee, 2009). Nitrates can be reduced to nitrite in the mouth and stomach by the nitrate‐reducing bacteria (Duncan et al., 1997). Nitrite is found widely in nature and is usually added to food as a preservative (Lu, Dong, Li, & He, 2016). Considering that the pH of the gastric lumen decreases to below 4 after eating 0.5–1 hr (Gardner et al., 2002), the results of the study suggested that the reactions of nitrite with polyphenols in pomegranate peel may be possible in the stomach (Figure 4). The rate constants in Table 1 show that α‐punicalagin and β‐punicalagin may react rapidly with nitrites. This reaction is because of its good water solubility. The inhibition of the formation of N‐nitrosoamines might be beneficial for humans.

**Figure 5:**
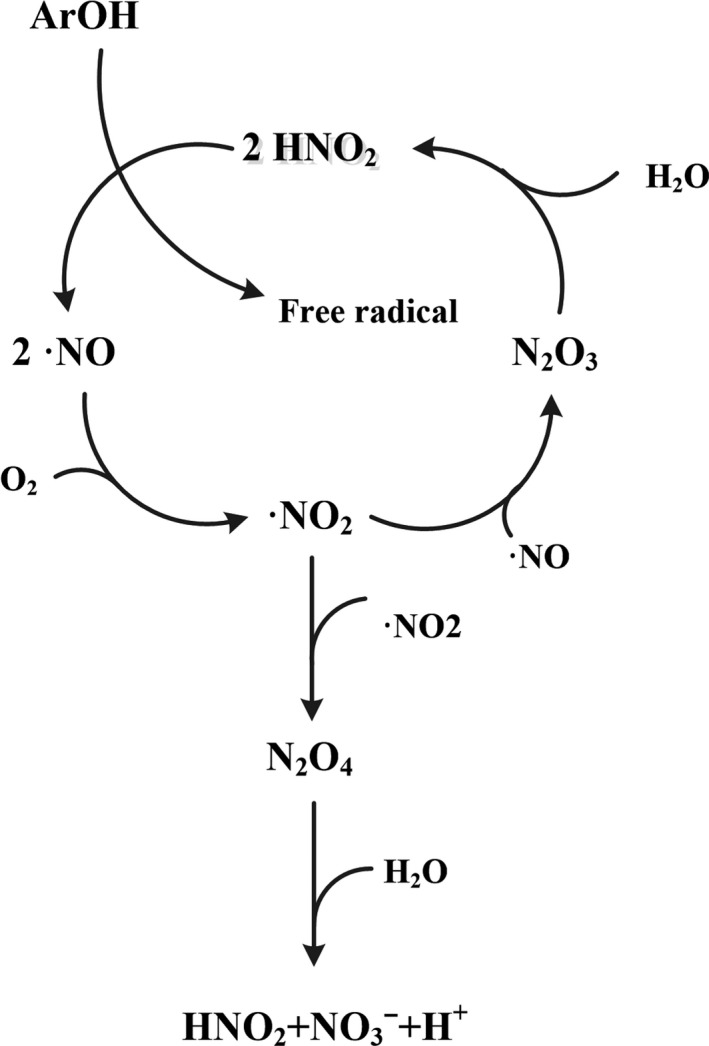
Chemical cycle of nitrite involved in phenolic compounds in acid solution

In recent years, the free radical reaction pathways of plant polyphenols and nitrites in acidic environments have been accepted and recognized by international academic circles. Previous studies by Peri et al. (2005) proved that •NO and •NO_2_ are formed in solutions containing acidic ${\text{NO}}_{2}^{-}$ (Figure 5). Nitration and nitrosation of polyphenols results from scavenging •NO and •NO_2_ free radicals. Semiquinone radicals were supposed to be the initial reaction products. As the fragment ions of (*m/z* 1055), punicalin moiety (*m/z* 781), and gallagic acid moiety (*m/z* 601) were found by ESI‐MS, the reaction is most likely to occur at position 1 and (or) 2 (Figure 1). Three adjacent phenolic hydroxyl groups on the benzene ring are unable to form quinones and cannot form a semiquinone radical.

## 5 CONCLUSIONS

This study showed that the polyphenols in pomegranate peel can react with nitrite under simulated stomach conditions. α‐punicalagin and β‐ punicalagin reacted more rapidly and easily with nitrite than ellagic acid. The reaction may be accompanied by the formation of three compounds, which were supposed to be isomers. The reactions of pomegranate peel polyphenols with nitrite at pH 2 react easily. As the pH value increased, the reaction decreased, and almost no reactions were observed at pH 4.5. This finding suggests that the reactions can occur in the stomach after a meal.

## CONFLICT OF INTEREST

The authors declare that they have no conflict of interest.

## ETHICAL APPROVAL

This study does not involve any human or animal testing.

## INFORMED CONSENT

Written informed consent was obtained from all study participants.
